# Superior gait performance and balance ability in Latin dancers

**DOI:** 10.3389/fmed.2022.834497

**Published:** 2022-08-24

**Authors:** Yen-Ting Liu, Ang-Chieh Lin, Szu-Fu Chen, Chih-Jen Shih, Tien-Yun Kuo, Fu-Cheng Wang, Pei-Hsin Lee, Adeline Peiling Lee

**Affiliations:** ^1^Department of Physical Medicine and Rehabilitation, Cheng Hsin General Hospital, Taipei, Taiwan; ^2^Department of Physiology and Biophysics, National Defense Medical Center, Taipei, Taiwan; ^3^Department of Mechanical Engineering, National Taiwan University, Taipei, Taiwan; ^4^Power and Health Physical Medicine and Rehabilitation Center, Taipei, Taiwan; ^5^Taipei Love Dance Company, Taipei, Taiwan

**Keywords:** gait, performance, symmetry, balance, stability, Latin dance, IMU, muscle

## Abstract

**Background:**

Latin dance consists of various fast and stability-challenging movements that require constant body adjustments to maintain proper posture and balance. Although human gaits are assumed to be symmetrical, several factors can contribute to asymmetrical behavior of the lower extremities in healthy adults. These include lower limb dominance, ground reaction forces, lower limb muscle power, foot placement angle, and range of joint motion. Gait impairment can lead to a high risk of falling, diminished mobility, and even cognition impairment. We hypothesized that Latin dancers might have a more symmetric gait pattern and better balance ability than healthy non-dancer controls.

**Methods:**

We investigated the impact of Latin dance training on gait behaviors and body balance. We recruited twenty Latin dancers and 22 normal healthy subjects to conduct walking experiments and one-leg stance tests, and we measured their kinematic data by inertial measurement units. We then defined four performance indexes to assess gait performance and body stability to quantify the potential advantages of dance training.

**Results:**

We found that the two gait asymmetric indexes during the walking test and the two performance indexes during the one-leg stance tests were better in Latin dancers compared with the healthy control group. The results confirmed the superiority of Latin dancers over the healthy control group in gait symmetry and balance stability. Our results suggest that Latin dancing training could effectively strengthen lower limb muscles and core muscle groups, thereby improving coordination and enhancing gait performance and balance.

**Conclusion:**

Latin dance training can benefit gait performance and body balance. Further studies are needed to investigate the effect of Latin dance training on gait and balance outcomes in healthy subjects and patients with gait disorders.

## Introduction

The maintenance of stable movements involves a complex interaction of the nervous, musculoskeletal, and cardiorespiratory systems (1). For this reason, gait can be a sensitive indicator of overall health and functional status, even in healthy adults without underlying pathological conditions (2). For example, walking speed has even been recommended as the “6th vital sign” (3). Gait impairment can lead to a high risk of falling (4), diminished mobility (5), and even cognition impairment. More than 30% of people over 65 years of age fall at least once a year because of loss of balance and muscle weakness (6). Thus, gait impairment may greatly affect the quality of life, functional independence, and health status (7). The observation of gait performance may therefore provide information on many factors, such as musculoskeletal condition, neurological status, dynamic balance, and postural control ability (8).

The mode and efficiency of a person’s gait depend on core and lower limb muscle strength, coordination, and free joint mobility (9, 10). Accumulating evidence suggests that exercise interventions, such as muscle strength, endurance, and balance training, can improve gait performance in healthy adults, especially in elderly subjects (11). Exercise can also help to reduce the rate of falls by approximately 25% and the number of older adults who experience falls by 15% (6). Dance is an easily accessible exercise that combines strength and coordination practice and provides high levels of enjoyment, thereby increasing exercise program adherence (12). Dance-based exercises can improve balance capability and gait performance in both young and old subjects (13, 14).

Latin dance is a type of ballroom dancing enjoyed by a part of the general population for its physical and psychological benefits. Latin dance consists of a complex dance sequence involving three-dimensional motions. Technical requirements include proper body posture, alignment, and rhythm which require an effort to maintain static and dynamic balance. The dance movement involves multiple segments of the body, with continuous alternations between one-leg and double-leg stance phases, such as hopping, sideways steps, or crossing one foot behind the other, thus requiring strong postural control. As a result, dancers become skilled at weight shifts that constantly challenge their postural control systems to maintain balance. This complex balance training enhances muscular strength, endurance, and coordination. It may also be a beneficial activity for gait stability since Latin dancing movements involve a complex motion of core muscles and lower limb muscles. The limited literature that has examined the effects of Latin dance indicates that Latin dance practice can improve mobility, strength, balance, and gait stability in older adults (15–17). However, information regarding the actual quantitative gait performance and balance ability of Latin dancers is lacking.

Human gait is a symmetrical and rhythmic periodic motion. A gait cycle typically consists of about 60% stance phase and 40% swing phase, and both legs are usually symmetrical in healthy persons. Therefore, gait performance can be assessed by the symmetry of leg movements. Many methods are available for obtaining gait information. For instance, Knutsson and Richards (18) applied electromyogram signals to identify muscle activation patterns. Optical motion capture systems, such as VZ4000 (19) and VICON (20), used high-speed cameras to record the positions of the markers attached to subjects. For example, Wang et al. (21) applied VZ4000 to detect stroke patients’ motions and automatically repeat the therapists’ interventive rehabilitation. Much research applied force plates to obtain gait information. For instance, Wong et al. (22) implemented load sensors to analyze foot contact patterns and evaluate walking abilities. Daliri (23) and El Maachi et al. (24) used the ground reaction forces underneath both feet to detect subjects with Parkinson’s disease (PD). However, the optical systems and the force plates can be costly, and their measurements are restricted to particular locations. In addition, the optical systems need to process markers’ position data into corresponding velocity and acceleration data. Therefore, Bilro et al. (25) used wearable optical sensors to monitor their subjects’ gaits. Díaz et al. (26) discussed the use of wearable sensor technologies in gait analysis and motion research. In the present paper, we applied inertial measurement units (IMUs) to measure kinematic data and to evaluate the influences of Latin dance training on gait behaviors and balance performance.

We recruited Latin dancers and age-matched healthy subjects as a non-dancer control group to conduct experiments. During the walking experiments, we attached two IMUs to these subjects’ limbs to obtain their gait information. We also conducted the one-leg stance tests and applied an IMU to the subjects’ waists to measure their body movements. The recorded IMU data were then used to evaluate the subjects’ gait performance and balance ability. The results showed that Latin dancers tended to have better gait performance and body stability compared to the normal control group.

## Materials and methods

### Experimental design and data collection

This study was approved by the Institutional Review Board of Cheng Hsin General Hospital, Taiwan [(876)110-22]. All gait behaviors and balance performance data were collected from participants who provided written informed consent.

We recruited 20 Latin dancers from a Latin dance studio and 22 age-matched healthy subjects as a non-dancer control group to conduct our experiments. Inclusion criteria of the dancers were the following: (a) overall good health; (b) between the ages of 20 and 60 years old; (c) practicing 3–6 days per week and at least 5 years of experience in Latin dance; (d) no limb or leg discrepancy; (e) no history of surgery on the lower limbs or spine; and (f) no history of musculoskeletal injury over the lower back in the past 6 months. The non-dancer control subjects were 22 age- and gender-matched healthy participants, who had no experience with dance training and were free from injury (no musculoskeletal injuries or neurological conditions). The non-dancer control subjects participated in regular exercise at a frequency of 2 or 3 times a week. Our subjects’ data are illustrated in Table 1. These subjects took walking and stability tests, and we measured their kinematic data to compare the gait performance and body balance between Latin dancers and non-dancer control subjects. For the walking experiments, we attached two IMUs to these subjects’ limbs to obtain their gait information. We also conducted the one-leg stance tests and applied an IMU to the subjects’ waists to measure their body movements. The recorded IMU data were then used to evaluate the subjects’ gait performance and balance ability.

**TABLE 1:** Basic data of the subjects.

Subject	Age	Gender	Height (cm)	Weight (kg)	BMI	Dancing experience (year)
D1	21	F	161	47	18.1	11
D2	35	F	166	51	18.5	10
D3	36	M	172	68	23	25
D4	29	F	161	51	19.7	6
D5	33	M	176	75	24.2	9
D6	28	F	161	50	19.3	10
D7	38	M	173	69	23.1	18
D8	25	F	162	53	20.2	10
D9	29	M	177	70	22.3	13
D10	27	M	173	70	23.4	14
D11	38	M	196	68	17.7	18
D12	30	M	176	71	22.9	19
D13	34	F	162	50	19.1	12
D14	26	M	175	60	19.6	10
D15	31	M	176	78	25.2	10
D16	35	F	158	48	19.2	9
D17	32	M	178	74	23.4	6
D18	42	F	162	51	19.4	21
D19	26	M	168	77	27.3	15
D20	27	F	156	49	20.1	14
						**Exercise experience**
N1	35	F	182	95	28.7	Basketball, weight training
N2	33	F	163	75	28.2	Aerobics
N3	39	M	167	68	24.4	Walking
N4	40	F	151	58	25.4	Jogging, weight training
N5	25	F	154	48	20.2	Walking
N6	23	M	165	60	22.0	Football, weight training
N7	44	F	162	58	22.1	Pilates
N8	25	M	177	95	30.3	Jogging, yoga
N9	28	M	172	78	26.4	Walking
N10	29	M	171	67	22.9	Walking
N11	29	F	160	53	20.7	Walking, weight training
N12	31	M	174	75	24.8	Basketball, weight training
N13	28	M	170	58	20.0	Basketball, jogging
N14	34	F	156	70	28.8	Waking, mountain climbing
N15	28	F	168	46	16.3	Walking
N16	38	M	186	115	33.2	Walking
N17	38	M	164	75	27.9	Walking
N18	40	M	173	67	22.4	Basketball, mountain biking
N19	38	M	175	64	20.9	Jogging, walking
N20	33	F	160	64	25.0	Jogging, walking
N21	36	F	150	60	26.6	Jogging, walking
N22	38	F	161	59	22.7	Jogging, walking

### Inertial measurement unit system

**FIGURE 1:**
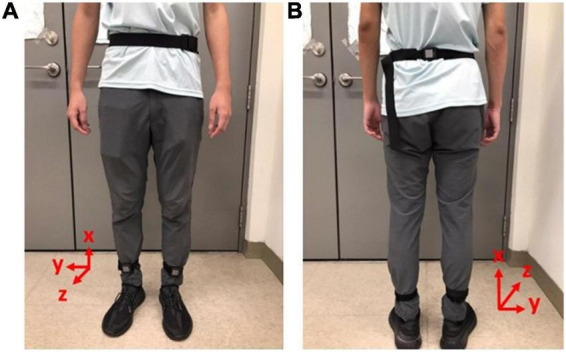
Gait measurements by IMUs. Two IMUs were attached to the subjects’ lower limbs and one IMU was attached to the subjects’ waist to record the subjects’ kinematic data with a sampling rate of 128 Hz. **(A)** Front view. **(B)** Rear view.

We applied the OPAL IMU system (27) with wearable IMUs to measure the kinematic data of these subjects during the experiments. The specifications of the OPAL IMU system are illustrated in Table 2. Two IMUs were attached to the subjects’ lower limbs and one IMU was attached to the subjects’ waist, as shown in Figure 1, to record the subjects’ kinematic data with a sampling rate of 128 Hz.

**TABLE 2:** Specifications of the OPAL IMU system (27).

Dimensions	43.7 × 39.7 × 13.7 mm
Weight	< 25 g (with battery)
Resolutions	17.5 bits
Sampling rates	20–128 Hz
Transmission range	30 m line of sight
Ranges of the accelerometer	± 200 g
Ranges of the gyroscope	± 2,000 deg/s
Ranges of the magnetometer	± 8 Gauss

### Evaluation of gait performance

**FIGURE 2:**
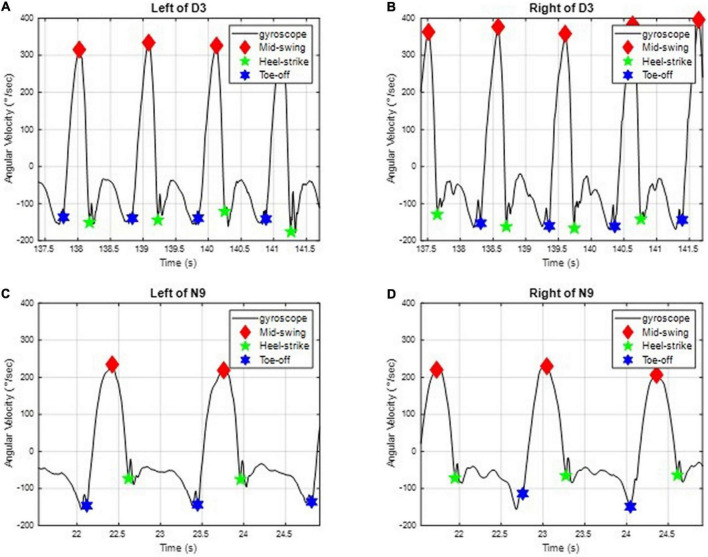
The angular velocities of D3 and N9 of the shank on the sagittal plane. The important gait events, such as the mid-swing, the heel strike, and the toe-off, are marked. **(A)** On the left leg of D3. **(B)** On the right leg of D3. **(C)** On the left leg of N9. **(D)** On the right leg of N9.

All subjects were required to walk in a straight line at their most comfortable pace. We applied two IMUs to measure the subjects’ angular velocities of the shanks on the sagittal plane (28) (i.e., the y-axis in Figure 1) to investigate the gait performance. For example, the angular velocities *ω*_y_ of D3 and N9 on the sagittal plane are shown in Figure 2, where the important gait events are marked. All subjects’ angular velocities are shown in Appendix 1.

**FIGURE 3:**
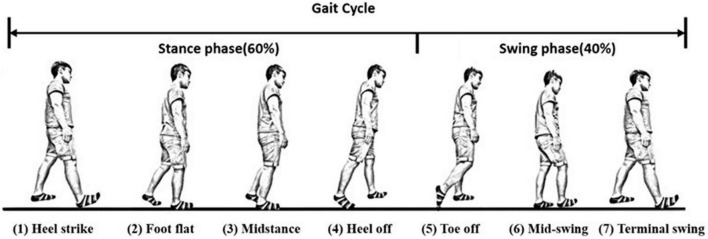
A complete gait cycle. A complete gait cycle is composed of a stance phase and a swing phase, containing three important gait events in one gait cycle: the mid-swing (MS), the heel strike (HS), and the toe-off (TO). The stance phase is defined as the time interval from the HS to the TO and accounts for about 60% of the gait cycle. The swing phase takes about 40% of the gait cycle and is defined as the time interval from the TO to the next HS.

We can further divide the gait responses into individual gait cycles, because human gaits are regular and periodic. A complete gait cycle is composed of a stance phase and a swing phase, containing three important gait events in one gait cycle: the mid-swing (MS), the heel strike (HS), and the toe-off (TO), as shown in Figure 3 (29). The MS occurs when the angular velocity of the shank on the sagittal plane reaches the maximum in the gait cycle; the HS event occurs when the heel touches the ground, where the angular velocity has the first negative trough after the mid-swing; and the TO occurs when the toes leave the ground (29). The stance phase is defined as the time interval from the HS to the TO and accounts for about 60% of the gait cycle. The swing phase is defined as the time interval from the TO to the next HS and takes about 40% of the gait cycle. Because gaits are periodic, a single gait cycle can be measured from any gait event to the next same event on the same foot. In this paper, we applied the IMU to measure gait information and used the angular velocity of the shanks on the sagittal plane to recognize these gait events (28, 30, 31). Because subjects might have varying walking speeds, we marked the HS events on the gait responses and divided the measured IMU data into individual gait cycles. All subjects’ gait cycles are illustrated in Appendix 2.

Differentiating the gait performance between dancers and the healthy controls from their gait cycles is difficult because of their similar appearance. Therefore, we proposed two indexes to quantify the gait performance of these subjects and to discuss the benefits of Latin dance training in improving gait performance. Healthy persons tend to have regular and periodic gaits, so the swing times of their two legs should be similar. Suppose the time interval of one complete gait cycle (from HS to HS) is represented as *T*_gait_ and the swing time (from TO to HS) of that gait cycle is *T*_*SW*_. The swing phase is then defined as the proportion of the swing time in the complete gait cycle, as in the following:


$$P_{SW}=\frac{T_{SW}}{T_{gait}}.$$

Therefore, we can define the asymmetry of the swing phases as follows: (29, 32)


$$Asym_{SP}=\frac{\left|{\left(P_{SW}\right)}_{R}-{\left(P_{SW}\right)}_{L}\right|}{\min\left\{\begin{array}{cc} {\left(P_{SW}\right)}_{R} & ,{\left(P_{SW}\right)}_{L} \end{array}\right\}}\times 100\%,$$

where (*P_SW_*)_R_ and (*P_SW_*)_*L*_ represent the swing phase on the right foot and the left foot, respectively. Gait symmetry has been assumed in healthy persons, and the swing time on both sides takes about 40% of the complete gaits. Therefore, the ideal *Asym_SP_* should be zero. However, gait asymmetry has been reported in healthy adults. For instance, asymmetrical behavior of the lower extremities was observed in spatio-temporal and kinematic parameters, such as lower limb dominance, ground reaction forces (33), electromyographic activities (34), lower limb muscle power (35), stride length (36), foot placement angle (37), and range of joint motion (38) in people without impairments. Clinically, gait asymmetry may be associated with a number of negative consequences, such as inefficient walking, impaired balance control, and increased risk of musculoskeletal injury to the dominant lower limb (39, 40). Apart from pathological conditions, such as stroke, amputation, or cerebral palsy, gait asymmetry is often observed in older adults and is a risk factor for falls (41). Hence, we can use *Asym_SP_* to evaluate the gait performance of our dancers and non-dancer subjects. All subjects’ asymmetries of swing phases *Asym_SP_* are illustrated in Appendix 3.

We can also define the ratio of swing time, denoted as *R*_*SW*_, as follows (29, 42):


$$R_{SW}=\frac{\max\left\{\begin{array}{cc} {\left(T_{SW}\right)}_{R} & ,{\left(T_{SW}\right)}_{L} \end{array}\right\}}{\min\left\{\begin{array}{cc} {\left(T_{SW}\right)}_{R} & ,{\left(T_{SW}\right)}_{L} \end{array}\right\}}\times 100\%,$$

where (*T_SW_*)_*R*_ and (*T_SW_*)_*L*_ represent the swing time on the right side and the left side, respectively. Gait in healthy adults has been assumed to be symmetric, so the swing time on both legs should be similar. Thus, the ideal value of *R*_*SW*_ should be 100% (43). Nevertheless, previous work has reported differences in gait parameters between the limbs (33–38). Therefore, we applied *R*_*SW*_ to evaluate the gait performance of these subjects. All subjects’ ratios of swing time are illustrated in Appendix 4.

Because the ideal value of *Asym_SP_* is zero and the ideal value *R*_*SW*_ is 100%, we were able to further quantify the gait performance by the following indexes:


$${\text{J}}_{1}={\left(\frac{1}{\text{N}}\sum_{i=1}^{\text{N}}{\left|{\text{Asym}}_{SP}\left(i\right)\right|}^{2}\right)}^{1/2},$$

$${\text{J}}_{2}={\left(\frac{1}{\text{N}}\sum_{i=1}^{\text{N}}{\left|{\text{R}}_{SW}\left(i\right)-100\right|}^{2}\right)}^{1/2},$$

where N is the number of gait cycles, and i represents the i-th gait cycle.

### Evaluation of balance ability

To investigate the balance ability, all subjects were required to conduct the following one-leg stance tests:

(a) standing on the dominant foot with eyes open;
(b) standing on the dominant foot with eyes closed;
(c) standing on the non-dominant foot with eyes open;
(d) standing on the non-dominant foot with eyes closed.

The subjects were asked to stand on one foot for about 50 s at each action if possible, but they were free to stand on both feet if body balance could not be maintained. They could take a rest of about 20 s between each action.

We implemented one IMU on the subjects’ waist to record their angular velocities and linear accelerations during the actions. When we stand on one foot, our bodies usually need to adjust position to maintain balance, like an inverted pendulum. Therefore, we were able to estimate the balance ability of these subjects from their kinematic data.

We defined the following two indexes to quantify the balance ability of the subjects:


$${\text{J}}_{3}=\frac{1}{\text{N}}\sum_{i=1}^{\text{N}}\left|\omega \left(i\right)\right|=\frac{1}{\text{N}}\sum_{i=1}^{\text{N}}{\left(\omega_{\text{x}}^{2}\left(i\right)+\omega_{\text{y}}^{2}\left(i\right)+\omega_{\text{z}}^{2}\left(i\right)\right)}^{1/2},$$

$${\text{J}}_{4}=\frac{1}{\text{N}}\sum_{i=1}^{\text{N}}\left|a\left(i\right)\right|=\frac{1}{\text{N}}\sum_{i=1}^{\text{N}}{\left({\text{a}}_{\text{x}}^{2}\left(i\right)+a_{\text{y}}^{2}\left(i\right)+a_{\text{z}}^{2}\left(i\right)\right)}^{1/2},$$

where N is the total number of samples, *ω*_x_(i), *ω*_y_(i), and *ω*_z_(i) are the three-axial angular velocities, and a_x_(i), a_y_(i), and a_z_(i) are the three-axial linear accelerations measured from the IMU on the waist at the i-th sample. That is, *ω*(i) and a(i) represent the absolute angular velocity and acceleration of the waist at sample i. Because subjects might have different balance durations, we defined the average absolute angular velocities and the average linear accelerations to estimate their balance ability. The subject’s balance ability is considered better if the average angular velocity and acceleration are smaller (i.e., the subject did not need much effort to maintain balance).

## Results

### Evaluation of gait performance

**FIGURE 4:**
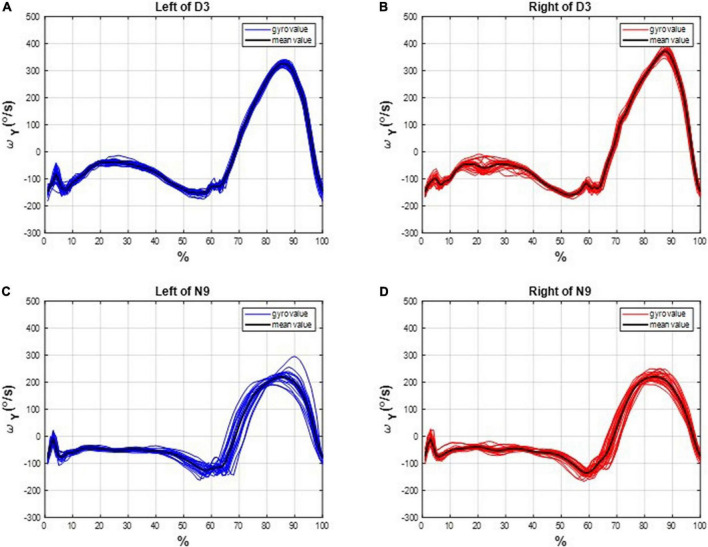
The gait cycles of D3 and N9. The dancer tended to have more uniform gait cycles than the non-dancer control, which indicates a superiority of dancing training on gait performance. **(A)** On the left leg of D3. **(B)** On the right leg of D3. **(C)** On the left leg of N9. **(D)** On the right leg of N9.

We marked the HS events on the subjects’ gait responses and divided the measured IMU data into individual gait cycles. For example, the gait cycles of D3 and N9 are shown in Figure 4, where the dancer D3 tended to have more uniform gait cycles than the non-dancer control N9.

**FIGURE 5:**
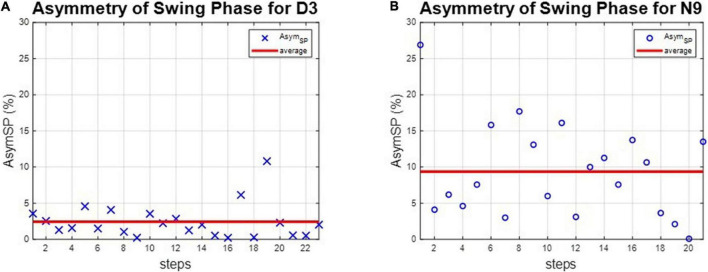
The asymmetry of the swing phases of D3 and N9. The dancer tended to have smaller *Asym_SP_* than the non-dancer control, because of dance training. **(A)** The dancer D3. **(B)** The non-dancer control N9.

**FIGURE 6:**
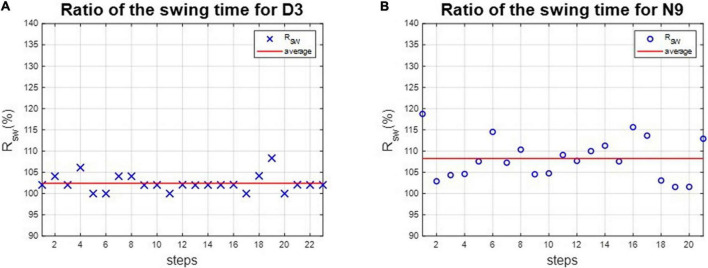
The ratio of swing time of D3 and N9. The dancer tended to have better *R*_*SW*_ than the non-dance control, because of dance training. **(A)** The dancer D3. **(B)** The non-dancer control N9.

The asymmetries of the swing phases *Asym_SP_* and the ratio of swing time *R*_*SW*_ were applied to evaluate the gait performance of dancers and healthy controls. For instance, the asymmetries of the swing phases of D3 and N9 are shown in Figure 5, while the ratio of swing time of D3 and N9 are shown in Figure 6. From Figures 5, 6, the dancer D3 tended to have better *Asym_SP_* and *R*_*SW*_ than the non-dancer control N9. All subjects’ asymmetries of swing phases *Asym_SP_* are illustrated in Appendix 3, while all subjects’ ratios of swing time are illustrated in Appendix 4.

**FIGURE 7:**
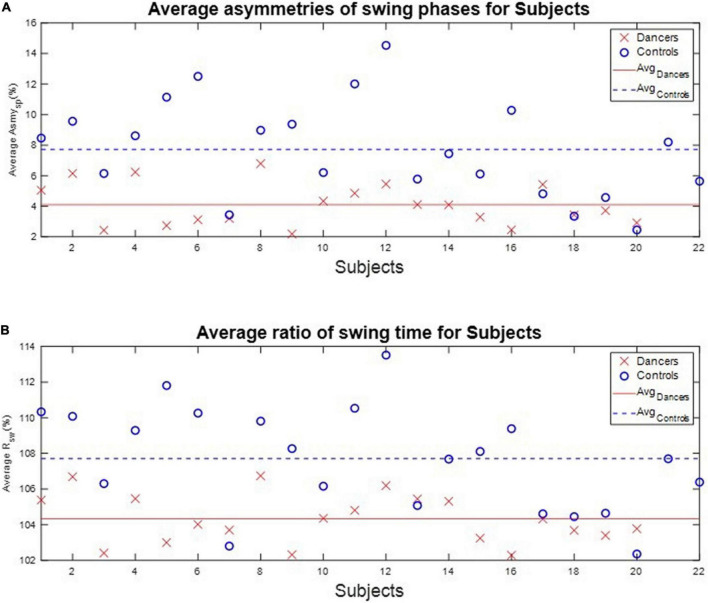
The average *Asym_SP_* and *R*_*SW*_ of subjects. The average *Asym_SP_* of the dancers ranged between 2.19% (D9) and 6.79% (D8), while the average *Asym_SP_* of the non-dancer controls ranged between 2.45% (N20) and 14.53 (N12). The average *R*_*SW*_ of dancers ranged between 102.29% (D16) and 106.74% (D8), while the average *R*_*SW*_ of healthy controls ranged between 102.35% (N20) and 113.52% (N12). The dancers tended to have better gait symmetry than the non-dancer controls. **(A)** The average asymmetry of the swing phases *Asym_SP_* of each subject. **(B)** The average ratio of swing time *R*_*SW*_ of each subject.

We calculated each subject’s average asymmetry of the swing phase *Asym_SP_* and average ratio of swing time *R*_*SW*_ in each gait cycle, as illustrated in Table 3 and Figure 7.

**TABLE 3:** Average *Asym_SP_* data and average *R*_*SW*_ for the test subjects.

Subject	Average *Asym*_SP_	Average *R*_*SW*_	J_1_	J_2_
D1	5.05	105.39	6.11	6.62
D2	6.15	106.69	7.42	8.11
D3	2.43	102.41	3.37	3.12
D4	6.24	105.46	7.32	6.98
D5	2.73	103.00	3.53	3.65
D6	3.12	104.02	4.40	4.84
D7	3.20	103.70	4.01	4.26
D8	6.79	106.74	7.89	7.82
D9	2.19	102.31	2.99	3.00
D10	4.33	104.36	5.31	5.04
D11	4.85	104.81	5.27	5.81
D12	5.46	106.19	6.63	7.89
D13	4.11	105.45	5.83	7.26
D14	4.09	105.31	6.61	7.56
D15	3.29	103.24	4.00	3.87
D16	2.46	102.29	3.22	2.76
D17	5.43	104.32	6.22	5.25
D18	3.44	103.68	4.52	4.93
D19	3.71	103.40	5.02	4.88
D20	2.92	103.78	4.10	4.96
N1	8.46	110.33	10.65	12.19
N2	9.57	110.08	12.47	13.80
N3	6.15	106.30	7.96	7.72
N4	8.62	109.29	10.11	11.54
N5	11.14	111.81	13.07	13.50
N6	12.50	110.26	13.97	11.78
N7	3.44	102.80	4.63	3.14
N8	8.97	109.81	12.42	13.26
N9	9.37	108.27	11.32	9.53
N10	6.21	106.16	6.45	6.51
N11	12.01	110.54	13.31	12.68
N12	14.53	113.52	16.65	15.63
N13	5.78	105.08	7.16	6.52
N14	7.44	107.68	9.96	9.61
N15	6.11	108.11	8.11	10.21
N16	10.28	109.39	10.49	10.23
N17	4.82	104.61	7.91	8.28
N18	3.35	104.45	4.43	6.94
N19	4.57	104.64	6.68	6.60
N20	2.45	102.35	3.27	3.24
N21	8.20	107.70	9.07	9.25
N22	5.64	106.39	8.49	8.69

First, the average *Asym_SP_* of the dancers ranged between 2.19% (D9) and 6.79% (D8), while the average *Asym_SP_* of the non-dancer controls ranged between 2.45% (N20) and 14.53 (N12). Second, the average *R*_*SW*_ of dancers ranged between 102.29% (D16) and 106.74% (D8), while the average *R*_*SW*_ of healthy controls ranged between 102.35% (N20) and 113.52% (N12). The dancers’ average *Asym_SP_* was closer to zero compared to the non-dancer control subjects, and the dancers’ average *R*_*SW*_ was closer to 100% compared to the non-dancer control subjects.

Table 3 also illustrates the two indexes, J_1_ and J_2_, to quantify the gait symmetry of the subjects. The results showed that, first, the average J_1_ is 5.19%, with a standard deviation of 1.51%, for the dancers. By contrast, for the non-dancer control group, the average J_1_ is 9.48% with a standard deviation of 3.39%. Second, the average J_2_ is 5.43%, with a standard deviation of 1.74%, for the dancers, while it is 9.58%, with a standard deviation of 3.32%, for the non-dancer control group. That is, the dancers tended to have better gait symmetry than the control subjects.

### Evaluation of balance ability

**FIGURE 8:**
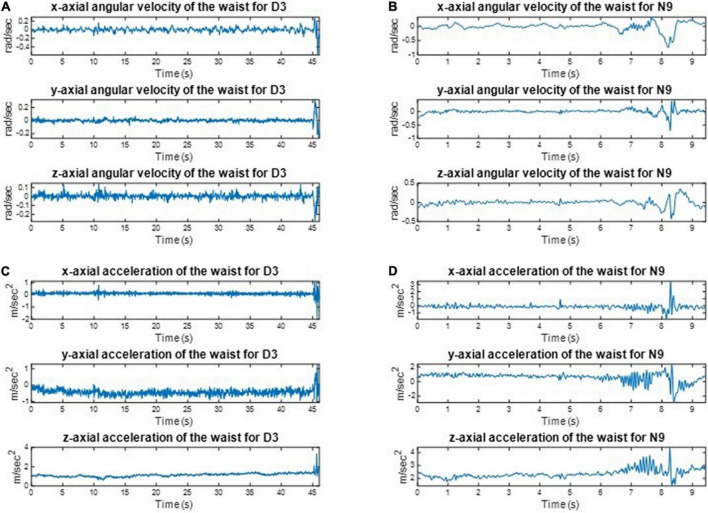
The recorded kinematic data of D3 and N9 when standing on the dominant foot with eyes open. The dancers tended to make smaller movements than the control group to maintain body balance. **(A)** The angular velocities of D3. **(B)** The angular velocities of N9. **(C)** The linear accelerations of D3. **(D)** The linear accelerations of N9.

**FIGURE 9:**
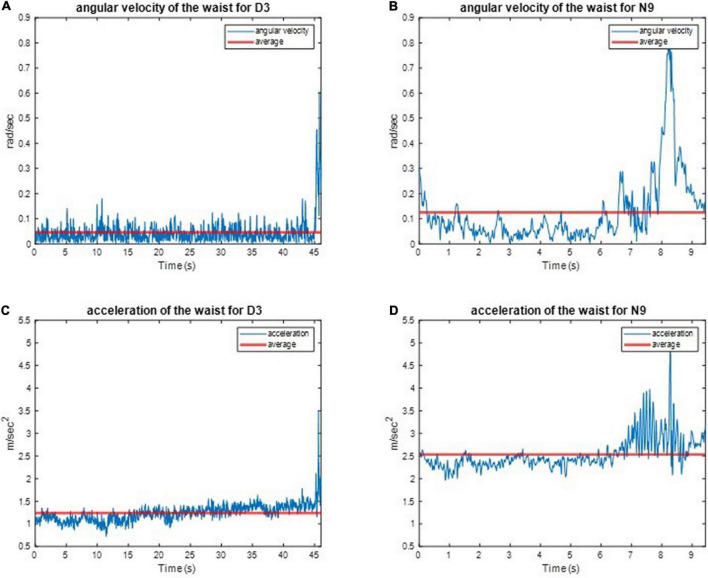
The absolute angular velocity and acceleration of D3 and N9 when standing on the dominant foot with eye open. The dancer made much smaller body movements than the non-dancer control maintain stability. **(A)** The absolute angular velocity of D3. **(B)** The absolute angular velocity of N9. **(C)** The absolute linear acceleration of D3. **(D)** The absolute linear acceleration of N9.

We implemented one IMU on the subjects’ waist to record their angular velocities and linear accelerations to estimate the balance ability of these subjects from their kinematic data during the one-leg stance tests. For example, the three-axial angular velocities and three-axial linear accelerations of D3 and N9, when standing on the dominant foot with eyes open (action (a)), are shown in Figure 8. All subjects’ IMU data during the one-leg stance tests are illustrated in Appendix 5. The dancers tended to make smaller movements than the control group to maintain body balance. The subject’s balance ability is considered better if the angular velocity and acceleration are smaller (i.e., the subject did not need much effort to maintain balance). For example, Figure 9 shows the absolute angular velocity *ω*(*t*) and the absolute linear acceleration a(t) of D3 and N9 when standing on the dominant foot with eyes open [action (a)]. Apparently, the dancer D3 made much smaller body movements than the non-dancer control N9 to maintain stability.

**FIGURE 10:**
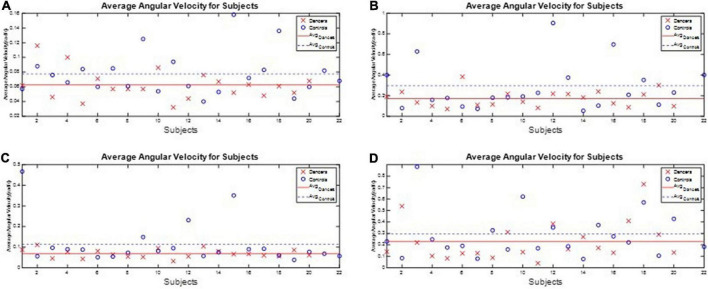
The performance index J_3_ during the one-leg stance tests. The dancers’ angular velocity index J_3_ were about 80.68, 58.52, 60.66, and 77.34%, respectively, of those of the healthy controls during the four actions. **(A)** Standing on the dominant foot with eyes open. **(B)** Standing on the dominant foot with eyes closed. **(C)** Standing on the non-dominant foot with eyes open. **(D)** Standing on the non-dominant foot with eyes closed.

**FIGURE 11:**
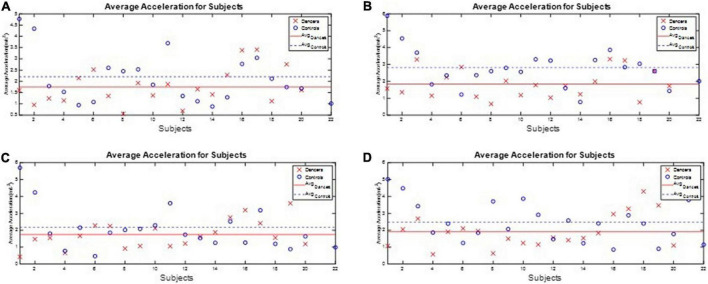
The performance index J_4_ during the one-leg stance tests. The dancers’ linear acceleration index J_4_ were about 79.39, 65.20, 80.18, and 77.61%, respectively, of the healthy controls during the four actions. **(A)** Standing on the dominant foot with eyes open. **(B)** Standing on the dominant foot with eyes closed. **(C)** Standing on the non-dominant foot with eyes open. **(D)** Standing on the non-dominant foot with eyes closed.

All subjects’ angular velocity and acceleration of the waist during the one-leg stance tests are illustrated in Appendix 6. The subjects’ average J_3_ and J_4_ during the one-leg stance tests are shown in Figures 10, 11, respectively, which show that the average J_3_ and J_4_ of the dancers were much lower than those of the normal controls. The statistical data of Figures 10, 11 are shown in Table 4. The dancers’ angular velocity index J_3_ were about 80.68, 58.52, 60.66, and 77.34%, respectively, of those of the healthy controls during the four actions. The dancers’ linear acceleration index J_4_ were about 79.39, 65.20, 80.18, and 77.61%, respectively, of the healthy controls during the four actions. That is, the dancer group needed less body adjustment to maintain postural stability.

**TABLE 4:** The performance indexes J_3_ and J_4_ of the subjects during the one-leg stance tests.

	J_3_				J_4_			
	(a)	(b)	(c)	(d)	(a)	(b)	(c)	(d)
**Dancers**								
D1	0.062	0.190	0.087	0.140	1.603	1.569	0.422	1.074
D2	0.116	0.237	0.111	0.538	0.954	1.354	1.472	2.052
D3	0.046	0.134	0.045	0.220	1.240	3.292	1.546	2.685
D4	0.100	0.100	0.077	0.103	1.146	1.146	0.648	0.573
D5	0.037	0.070	0.043	0.082	2.145	2.244	1.660	1.904
D6	0.071	0.384	0.081	0.126	2.522	2.846	2.277	2.100
D7	0.057	0.110	0.064	0.127	1.350	1.093	2.247	1.949
D8	0.057	0.115	0.054	0.087	0.554	0.663	0.916	0.625
D9	0.057	0.218	0.052	0.311	1.924	2.023	1.063	1.499
D10	0.086	0.141	0.095	0.137	1.373	1.178	2.109	1.237
D11	0.032	0.081	0.032	0.039	1.874	1.781	1.049	1.153
D12	0.044	0.219	0.055	0.384	0.690	1.031	1.205	1.554
D13	0.076	0.216	0.104	0.163	1.650	1.732	1.614	1.409
D14	0.067	0.182	0.080	0.270	1.416	1.234	1.880	1.531
D15	0.052	0.240	0.066	0.173	2.283	1.993	2.752	1.833
D16	0.063	0.125	0.067	0.131	3.382	3.315	3.192	2.959
D17	0.048	0.087	0.060	0.409	3.409	3.239	2.412	3.270
D18	0.061	0.211	0.057	0.730	1.121	0.762	1.560	4.301
D19	0.052	0.301	0.086	0.290	2.758	2.609	3.596	3.473
D20	0.068	0.098	0.061	0.133	1.611	1.723	1.183	1.097
Average	0.063	0.173	0.069	0.230	1.750	1.841	1.740	1.913
**Healthy controls**								
N1	0.057	0.400	0.467	0.230	4.773	5.881	5.704	5.023
N2	0.088	0.079	0.056	0.084	4.341	4.543	4.239	4.485
N3	0.076	0.629	0.097	0.882	1.787	3.699	1.800	3.426
N4	0.066	0.159	0.090	0.248	1.529	1.820	0.774	1.864
N5	0.084	0.177	0.088	0.177	0.945	2.348	2.151	2.389
N6	0.060	0.094	0.051	0.191	1.081	1.217	0.462	1.242
N7	0.085	0.072	0.054	0.079	2.598	2.364	1.852	1.847
N8	0.061	0.180	0.073	0.327	2.451	2.604	2.013	3.708
N9	0.125	0.183	0.149	0.160	2.534	2.794	2.076	2.069
N10	0.054	0.193	0.081	0.621	1.849	2.563	2.294	3.869
N11	0.094	0.227	0.095	0.170	3.695	3.306	3.601	2.913
N12	0.061	0.905	0.231	0.352	1.348	3.231	1.736	1.473
N13	0.040	0.375	0.057	0.187	1.115	1.595	1.537	2.576
N14	0.053	0.053	0.075	0.076	0.881	0.775	1.253	1.229
N15	0.158	0.103	0.351	0.373	1.291	3.264	2.516	2.402
N16	0.072	0.697	0.090	0.274	2.773	3.865	1.266	0.850
N17	0.083	0.208	0.092	0.222	3.046	2.840	3.181	2.885
N18	0.136	0.352	0.060	0.571	2.119	3.039	1.191	2.396
N19	0.044	0.112	0.038	0.105	1.745	2.606	0.879	0.897
N20	0.060	0.230	0.077	0.427	1.687	1.433	1.637	1.775
N21	0.082	0.673	0.068	0.594	3.898	4.331	4.600	3.793
N22	0.068	0.401	0.057	0.183	1.015	2.009	0.986	1.145
Average	0.078	0.296	0.114	0.297	2.204	2.824	2.170	2.466

## Discussion

### Evaluation of gait performance

In this paper, we assess gait performance by the symmetry of gaits. Gait symmetry has been assumed in healthy persons, and the swing time on both sides takes about 40% of the complete gaits. Therefore, the ideal asymmetry of the swing phase *Asym_SP_* should be zero and the ideal ratio of swing time *R*_*SW*_ should be 100%. However, as we described above, gait asymmetry has been reported in healthy adults (33–38). Clinically, gait asymmetry may be associated with a few negative consequences, such as inefficient walking, impaired balance control, and increased risk of musculoskeletal injury to the dominant lower limb (39, 40). Gait asymmetry is often observed in older adults and is a risk factor for falls (41). Hence, *Asym_SP_* and *R*_*SW*_ can be applied to evaluate the gait performance of the dancers and non-dancer subjects. The average *Asym*_*SP*_ was 4.10% for dancers, but 7.71% for healthy controls, while the average *R*_*SW*_ was 104.33% for dancers, but 107.71% for healthy controls. Our results showed that the asymmetries of the swing phases of dancers are smaller than those of non-dancer controls. Latin dance consists of a complex dance sequence involving motion of core and hip muscles. During dancing, core muscles, including the transverse abdominis, multifidus, rectus abdominis, and erector spinae, work in coordination to maintain proper body posture and alignment. The unique hip motion, characterized by a rhythmic rotation of the hips around the spine, is essential for Latin dance, and the core and hip muscles work sequentially and symmetrically. In addition, as Latin dancing is predominantly based on walking actions, repeatedly performing dance steps works the major thigh muscles involved in walking, such as the quadriceps and hamstring muscles. Our data imply that the Latin dancing might improve gait symmetry through the enhancement of dynamic postural control and the strengthening of the core and lower limb muscles. Another possible reason for the Latin dancers’ gait superiorities is the body awareness required in Latin dancing. Previous work has demonstrated that ballroom dancing can provide benefits to body perception (44). Latin dancing is a complex sensorimotor activity that integrates many skills, such as rhythm, synchronization movements with music, balance, coordination, and spatial sense. Dancers need to perceive their anatomical body space and the planes of movements, and then interact precisely *via* integration of tactile, proprioceptive, kinesthetic, and environmental information. Thus, Latin dancers may direct body awareness toward greater positional body symmetry during walking.

Among the non-dancer controls, N7, N18, and N20 had better gait symmetry compared with the others, possibly because of their regular exercise programs. For example, subject N7 has been practicing Pilates regularly for more than 10 years. Pilates is a combination of stretch and strengthening exercises performed in a neutral spine position (45). Through stretching and isometric, eccentric, and concentric muscle strengthening, Pilates improves balance skills, proprioception, coordination, and postural control. Therefore, Pilates exercises can also improve various motor abilities and are effective in enhancing postural stability, dynamic balance, flexibility, muscle strength, and walking capabilities (46, 47). Subject N18 is a mountain biker who has been riding mountain bikes regularly for more than 10 years. Mountain biking is a dynamic activity that requires participants to constantly change their body position to adapt to the bumpy road (48). Both the abdominal and core muscles play an important role in this form of exercise to maintain good control of the bike, so that mountain bikers have better balance and coordination skills compared with the general population. Subject N20 conducts regular walking and jogging, which are similar to the activities of the other control participants. The regimen of jogging and walking, genetic factors, or other environmental factors, such as nutrition, may possibly play a role in maintaining gait symmetry.

### Evaluation of balance ability

The one-leg stance is a frequently used clinical tool for assessment of balance in persons with various balance disorders (49). The subject’s balance ability is considered better if the angular velocity and acceleration are smaller (i.e., the subject did not need much effort to maintain balance). We found that the dancers’ linear acceleration index J_4_ were about 79.39, 65.20, 80.18, and 77.61%, respectively, of the healthy controls during the four actions. That is, the dancer group needed less body adjustment to maintain balance. Our results showed that Latin dancers had smaller trunk movements while conducting the one-leg stance test, indicating that Latin dancers have better postural steadiness compared with the non-dancer controls. As mentioned above, Latin dancers tend to have stronger core muscle strength, which plays a crucial role in maintaining both posture and trunk stability. In contrast to gait asymmetry, the one-leg stance is an easy clinical balance test that can assess a person’s postural steadiness in a relatively static condition. Our finding of improved leg stance in Latin dancers is important clinically, since poor one-leg balance is a significant predictor of injurious falls, especially in the elderly population (50). Vision also plays a crucial role in processing and integrating other sensory information involving postural control and balance (51). We avoided the impact of visual feedback by conducting the one-leg stance test with eyes both closed and open. The dancers had significantly better balance with one-leg standing with eyes open, as a less challenging level, but they also had better balance with eyes closed, as a more challenging level, through improved proprioception. That is, the Latin dancers could still maintain better postural stability without visual dependence. Our findings suggest that Latin dancing is a promising exercise training for fall prevention.

However, this study has some limitations. One was that only a small number of Latin dancers and non-dancer healthy participants were recruited, so the data may not be generalizable to Latin dancers at large. Another was that our study design was a cross-sectional observational study, so further interventional research is necessary to clarify the efficacy of Latin dance training. A further limitation is that although we recruited non-dancer controls who were physically active and exercised regularly, the physical activity was reported by questionnaires and the exercise types differed significantly among participants. Further studies should focus on participants who engage in the same sports to provide a better comparison with Latin dancers. In the future, we plan to collect more kinematic data and to consider other performance indexes to investigate the potential advantages of Latin dance training in improving postural stability, balance, and gait performance in patients with neurological or orthopedic disorders.

## Conclusion

This paper investigated the potential benefits of Latin dance training on gait behaviors and balance ability. Several factors, such as lower limb dominance, ground reaction forces, lower limb muscle power, foot placement angle, and range of joint motion, can contribute to asymmetrical behavior of the lower extremities in people without impairments. Because Latin dancing movements involve a complex motion of core muscles and lower limb muscles, this type of exercise may be a beneficial activity for improved gait stability. In this paper, we recruited twenty dancers and twenty-two age-matched normal healthy subjects to conduct walking experiments and one-leg stance tests. Their gait responses and body movements were recorded by IMUs and then used to evaluate gait performance and body stability. We defined four performance indexes to quantify gait performance and balance ability. The results showed that Latin dancers had better gait symmetry and body balance than the healthy control group, suggesting that gait performance and stability might be improved by Latin dance training. The possible contributing factors are enhancing dynamic postural control, strengthening the core and lower limb muscles, and better body awareness. Further studies are needed to investigate the effect of Latin dance training on gait and balance outcomes in healthy subjects and patients with gait disorders. This paper applied the commercial OPAL IMU system because of its high sampling rates and resolutions. In the future, the smartphone IMU system might be compatible as technologies advance and can be used for performance evaluation in clinical practice.

## Data availability statement

The original contributions presented in the study are included in the article/supplementary material, further inquiries can be directed to the corresponding author/s.

## Ethics statement

The studies involving human participants were reviewed and approved by his study was conducted according to the guidelines of the Declaration of Helsinki, and approved by the Institutional Review Board of Cheng Hsin General Hospital, Taiwan [no. (876)110-22; date of approval: 7 Oct, 2021]. Informed consent was obtained from all subjects involved in this study. The patients/participants provided their written informed consent to participate in this study.

## Author contributions

Y-TL, S-FC, and F-CW contributed to conception and design of the study. Y-TL, A-CL, C-JS, T-YK, P-HL, and AL contributed to experiments and data collection. C-JS and T-YK contributed to software development. C-JS, T-YK, and F-CW conducted data analyses. Y-TL, S-FC, C-JS, and F-CW wrote the first draft of the manuscript. All authors contributed to manuscript revision, read, and approved the submitted version.

## Appendix

Appendix 1

The angular velocities *ω*_y_ of all subjects are available at http://140.112.14.7/∼sic/PaperMaterial/Dance_Gait_Responses.zip.

Appendix 2

The gait cycles of all subjects are available at http://140.112.14.7/∼sic/PaperMaterial/Dance_IMU_Data.zip.

Appendix 3

The asymmetry of swing phases (*Asym*_*SP*_) of all subjects are available at: http://140.112.14.7/∼*sic*/PaperMaterial/Dance_Asym_Data.zip.

Appendix 4

The ratio of swing time (*R*_*SW*_) of all subjects are available at: http://140.112.14.7/∼*sic*/PaperMaterial/Dance_Ratio_Data.zip.

Appendix 5

The IMU data on waist of all subjects are available at: http://140.112.14.7/∼*sic*/PaperMaterial/Dance_Gait_Responses.zip.

Appendix 6

The angular velocity and acceleration of the waist for all subjects are available at: http://140.112.14.7/∼*sic*/PaperMaterial/Subjects’ angular velocity and acceleration of the waist.zip.
